# Death of Dr. Perrine

**Published:** 1840-10

**Authors:** 


DEATH OF DR. PERRINE.

The readers of this Journal, and of its predecessor in Cincinnati, the Western Journal of the Medical and Physical Sciences, will regret to hear that Dr. Henry Perrine, whose interesting communications have often met their eye, is no more. He was killed by the Indians during the night of the 6th of August last, at Indian Key, South Florida, where his last communication to the Journal was dated.

Dr. P. was a native of New Jersey, and received his professional education, we believe, in one of the New York schools. The commencement of his professional career was in the state of Indiana, whence he removed to Mississippi. In both states he distinguished himself by the successful energy of his practice in the malignant intermittents, which from fifteen to twenty years ago prevailed in the West and South. Accounts of his treatment of such cases, by scruple and even drachm doses of Sulphate of Quinine, were published soon afterwards in the American and the Western Journals. While residing in the state of Mississippi, he received the appointment of Consul to the Mexican city of Campeachy, in the province and peninsula of Yucatan, where, during a residence of several years, he made the natural productions and diseases of that region a subject of special study. When the epidemic Cholera reached it, his superior skill and benevolence rendered him a public benefactor. His account of the disease was published soon afterwards in the Western Journal.

During his residence abroad, his active and patriotic mind conceived the design of transplanting into the southern part of Florida a great number of tropical plants, useful in medicine, dietetics, and the arts. To accomplish this, he returned to the United States, and presented to Congress a memorial, praying for the grant of two townships of land in South or Tropical Florida, on which to make the experiment. A report in favor of his application was made, but not acted upon at that session. With indomitable energy, he pressed into his service a great number of his brethren and other scientific men, in different parts of the Union, and at the next or a subsequent session, new and elaborate reports in favor of the object having been made to both Houses of Congress, he obtained the grant of one township.

Although his constitution had been greatly injured by a residence in hot climates, he repaired forthwith to the scene of his future operations, and entered upon them, with his characteristic zeal. He had fixed his residence with a few families at Indian Key, which in the darkness of the night was attacked by 100 or 150 Indians. He went to the cupola of his house and spoke to them in the Spanish language, when he was shot, or at least did not return. His wife and three children effected their escape. His house was burned up, and all his valuable notes and papers, on the production and diseases of the South consumed along with his body. The following tribute to his character, we extract from a letter of the Hon. James Whitcomb, Commissioner of the General Land Office, at Washington, dated August 24th:

"I enclose you this morning's Intelligencer, by which you will see, that our mutual friend, Dr. H. Perrine has fallen a victim to the savages of Florida. Besides the irreparable loss to his family and his friends, I am apprehensive that his untimely death will defeat the enterprize to which he had, at a considerable expense and a great loss of time devoted himself for the cultivation of tropical plants in that territory. Congress had at the session before the last, as I presume you are apprized, granted to them a tract of land for that purpose, and orders were issued, at his request, not long since, from the General Land Office for the survey of the grant.

"Philanthropy with him was a moving and active principle, nay it was carried to the verge of enthusiasm. He looked, I doubt not upon the Seminoles, as misguided, but well-meaning, and that he could successfully appeal to their humanity. Hence his attempt at a parley and his melancholy fate."

In several letters received at different times, Dr. Perrine has sought to impress on us his own conviction, that Tropical Florida is actually one of the healthiest spots on the continent, and peculiarly fitted by its climate, for the winter residence of the consumptive.

